# Comparative Proteomic Analysis of the Mitochondria-associated ER Membrane (MAM) in a Long-term Type 2 Diabetic Rodent Model

**DOI:** 10.1038/s41598-017-02213-1

**Published:** 2017-05-18

**Authors:** Jacey Hongjie Ma, Shichen Shen, Joshua J. Wang, Zhanwen He, Amanda Poon, Jun Li, Jun Qu, Sarah X. Zhang

**Affiliations:** 1 Department of Ophthalmology and Ross Eye Institute, University at Buffalo, State University of New York, Buffalo, NY USA; 2 SUNY Eye Institute, State University of New York, New York, NY USA; 3 Aier School of Ophthalmology, Central South University, Changsha, China; 4 Department of Biochemistry, University at Buffalo, State University of New York, Buffalo, NY USA; 5 New York State Center of Excellence in Bioinformatics and Life Sciences, 701 Ellicott Street, Buffalo, NY USA; 6 Department of Pediatrics, Sun Yat-Sen Memorial Hospital, Sun Yat-sen University, Guangzhou, China; 7 Department of Pharmaceutical Sciences, School of Pharmacy and Pharmaceutical Sciences, University at Buffalo, State University of New York, Buffalo, NY USA

## Abstract

The mitochondria-associated ER membrane (MAM) plays a critical role in cellular energetics and calcium homeostasis; however, how MAM is affected under diabetic condition remains elusive. This study presented a comprehensive proteome profiling of isolated brain MAM from long-term type 2 diabetic mice vs. non-diabetic controls. MAM protein was extracted efficiently by a surfactant-aided precipitation/on-pellet digestion (SOD) method, and MAM proteome was quantified by an ion-current-based MS1 method combined with nanoLC-MS/MS. A total of 1,313 non-redundant proteins of MAM were identified, among which 144 proteins were found significantly altered by diabetes. In-depth IPA analysis identified multiple disease-relevant signaling pathways associated with the MAM proteome changes in diabetes, most significantly the unfolded protein response (UPR), p53, hypoxia-related transcription factors, and methyl CpG binding protein 2. Using immunofluorescence labeling we confirmed the activation of three UPR branches and increased ERp29 and calreticulin in diabetic retinas. Moreover, we found GRP75, a key MAM tethering protein, was drastically reduced by long-term diabetes. *In vitro*, acute high glucose treatment reduces ER-mitochondrial contact in retinal endothelial cells. This study provides first insight into the significant alterations in MAM proteome associated with activation of the UPR in diabetes, which may serve as novel benchmarks for the future studies of diabetic complications.

## Introduction

Diabetes mellitus (DM) has emerged as a pandemic metabolic disorder worldwide. Currently, approximately 21.3 million Americans are suffering from diabetes and 1.9 million new cases are diagnosed each year^1^. Type 2 diabetes (T2DM), accounting for 90–95% of all cases, is the most prevalent form of diabetes and is associated with insulin resistance, hyperlipidemia, obesity, and hypertension. Chronic hyperglycemia and confounding risk factors lead to irreversible systemic complications particularly affecting cardiovascular system, kidney, retina, peripheral nerves and the brain. In the retina, diabetes damages both small blood vessels and neurons resulting in slowly developed neurovascular degeneration, macular edema, retinal ischemia, and uncontrolled new vessel growth in late stage^2^. Comparatively, damage to the brain by diabetes is less well defined in the past but is gaining increasing attention^3^. Clinical studies show that T2DM patients have higher risk of decline in cognitive functions, decrease in brain volume, and developing ischemic or hemorrhagic brain lesions^4, 5^. The exact mechanisms underlying the structural abnormalities and functional defects in diabetic brain and retina remain elusive.

Recent studies have established that disturbed homeostasis of the endoplasmic reticulum (ER), or ER stress, and mitochondrial dysfunction are critically involved in neurovascular injury and cell death in diabetes and neurodegenerative diseases^6, 7^. The ER and mitochondrial dysfunction often co-exist and this intrinsic link can be attributed to the physical interactions between the two organelles via the mitochondria-associated ER membrane (MAM). Despite its discovery over 40 years ago, the importance of MAM in cellular metabolism and signaling has not been recognized until recent years^8^. In vertebrates, the formation of MAM is dynamically regulated by proteins including: inositol 1, 4, 5-triphosphate receptor (IP3R; ER side), glucose-regulated protein 75 (GRP75), and voltage-dependent anion channel 1(VDAC1; mitochondria side)^9^, mitofusin-2, and phosphofurin acidic cluster sorting protein-2 (PACS2)^10^. The MAM contains chaperones, oxidoreductases, calcium channels and buffering proteins, as well as regulators of lipid metabolism. Thus, this subcellular compartment is likely involved in cell metabolism by orchestrating protein folding, lipid synthesis, calcium buffering^11^ and oxidation/reduction^12, 13^. Perturbation of MAM function reduces mitochondrial ATP production, increases reactive oxygen species (ROS) generation, and exacerbates ER stress resulting in apoptosis^14, 15^. These findings imply a potential role of MAM in the development of diabetic complications, yet how diabetes influences the structure, signaling and functions of MAM has not been studied.

The *Lepr* ^*db*^ (*db/db*) mouse model recapitulates many clinical features of human T2DM, including obesity, hyperglycemia, insulin resistance and hyperlipidemia. Here, we employed 15 month-old *db/db* mice to investigate the molecular alterations of MAM in long-term diabetes using a sensitive and reproducible nanoLC-MS/MS combined with a novel ion-current-based MS1 method. Based on the quantitative results we conducted bioinformatic analysis to identify disease-relevant signaling pathways implicated by the changes of the MAM proteome patterns, and proteins and pathways of high interest were examined using immunofluorescence labeling.

## Results

### Isolation and Verification of MAM from *db/db* and *db*/+ Mice

**Figure 1:**
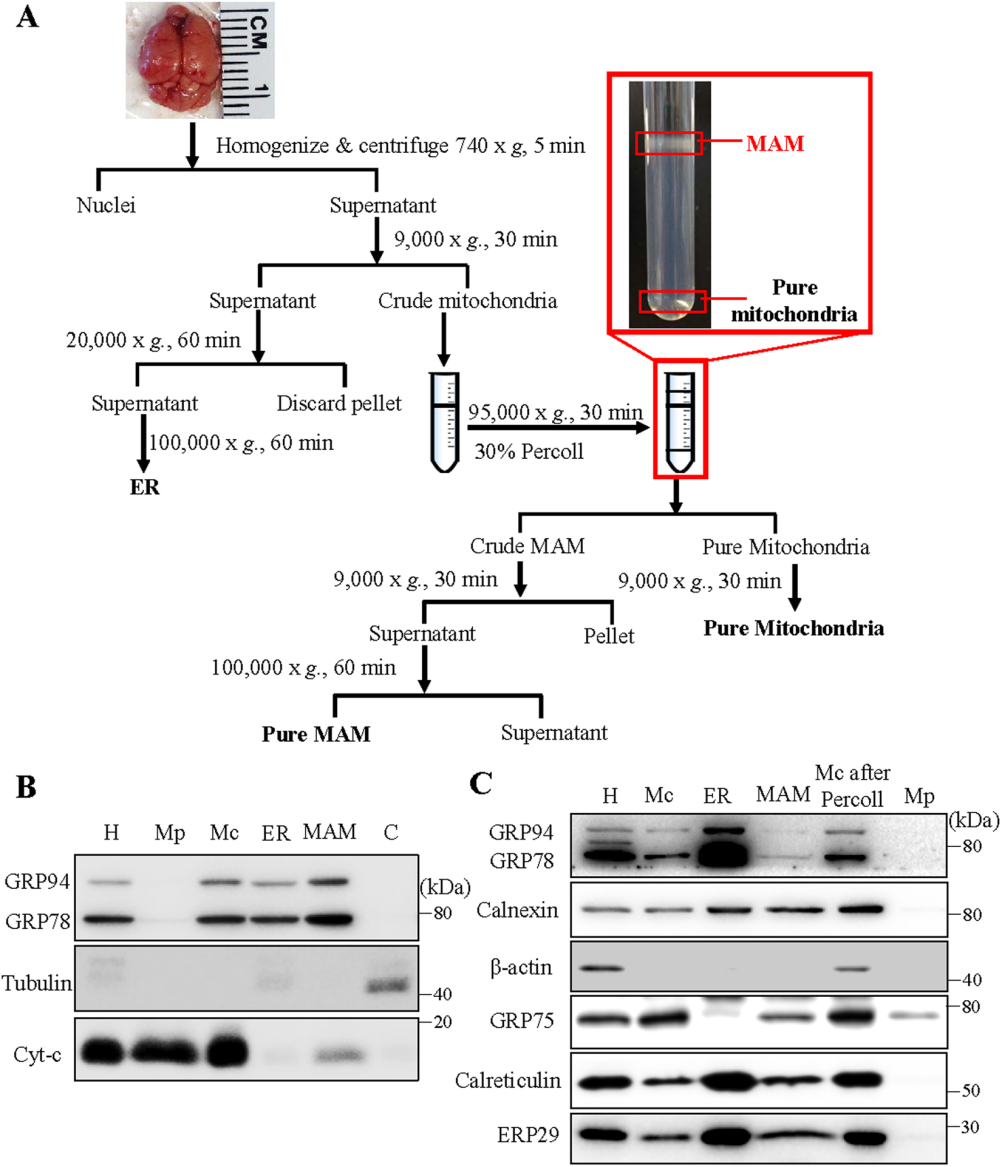
Schematic diagram of MAM isolation and confirmation of MAM associated proteins by western blotting. (**A**) MAM was isolated from mouse brain by applying differential centrifugations and a self-forming Percoll gradient centrifugation. Other cell organelles, as crude mitochondria, pure mitochondria and ER, were also obtained following the multiple centrifuge steps. (**B,C**) Western blot analysis of organelle markers in isolated MAM from the brain (**B**) and retina (**C**) were enriched for KDEL, and free from tubulin and cytochrome-C contamination. H: homogenate, Mp: pure mitochondria, Mc: crude mitochondria, ER: endoplasmic reticulum, MAM: ER mitochondria-associated membrane, C: cytosol, Mc after percoll: crude mitochondria after percoll gradient centrifuge.

At the age of 15 months, *db*/*db* mice show increased blood glucose levels compared to *db/*+ controls (463 ± 44.29 *vs*. 166.8 ± 21.80 mg/dl, p < 0.01, n = 5) and body weights (52.17 ± 10.10 *vs*. 40.88 ± 5.73 g, P = 0.061, n = 5). Interestingly, the absolute weight of brain from *db/db* mice is significantly lower (378.4 ± 24.70 *vs*. 437.6 ± 18.23 mg, P < 0.01, n = 5), as is the brain to body weight ratio (0.747 ± 0.154 vs. 1.104 ± 0.103%, P < 0.01, n = 5), compared to *db/*+ mice. This finding is consistent with clinical observations of decreased brain volume in diabetic patients^4, 5, 16^. Further, histological analyses from previous studies show age-dependent cortical atrophy, hippocampal thinning, and apoptosis of retinal neurons in *db/db* mice^17, 18^. This suggests that long-term diabetes can impair the central nervous system resulting in neurodegeneration of brain and retina. To determine whether MAM is involved in the process of diabetes-induced neurodegenerative process, we isolated the MAM from *db/db* mice following a well-documented protocol summarized in Fig. 1A ^19, 20^. Western blot analysis shows that the MAM was enriched for glucose-regulated protein 78 (GRP78) and glucose-regulated protein 94 (GRP94) but devoid of gross contamination from cytosol (tubulin) and mitochondria (Cytochrome-C), indicating the high purity of MAM (Fig. 1B). Using the same method, we isolated MAM from retinal tissue pooled from 20 C57/BL6 mouse retinas. Western blot analysis verified the expression of calnexin, Calreticulin^21^ and GPR75^22^ in the MAM (Fig. 1C).

### Comprehensive and Quantitative Profiling of MAM Proteomes

**Figure 2:**
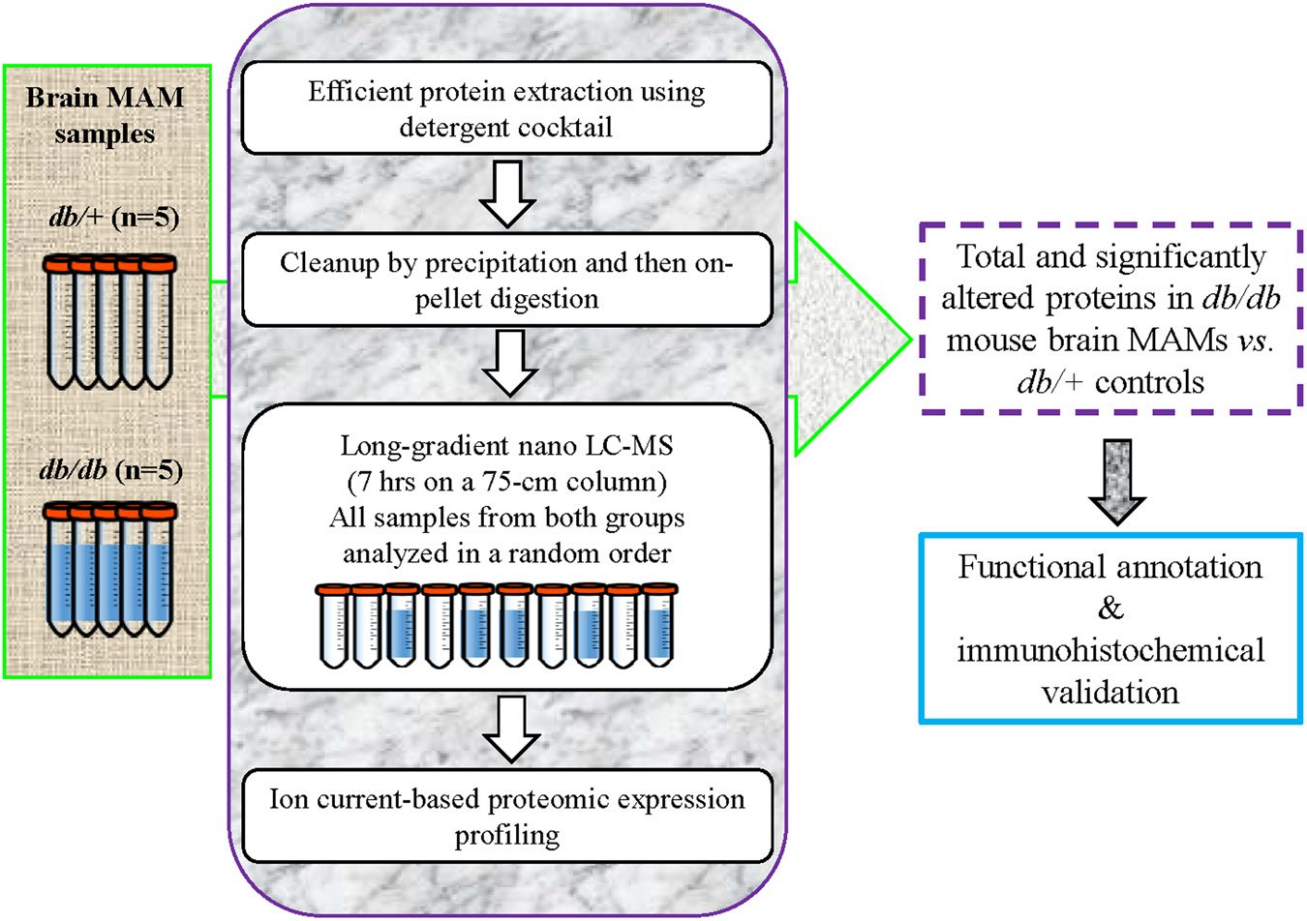
Scheme of proteomics strategy applied to analysis of mouse brain MAM samples from *db/db* mice *vs*. age- and gender-matched *db/*+ mice. A highly reproducible and extensive ion-current-based quantification method, known as long gradient nano-reverse-phase liquid chromatography/mass spectrometry, was applied in the analysis of the 5 biological replicates. David bioinformatics database (6.7, NIAID/NIH) and Ingenuity Pathway Analysis (IPA) were used for the function annotation of the identified proteins. The pathways and proteins of high interests were verified by immunohistochemical studies in the retina.

Because of the limited amount of retinal MAM, we used brain MAM samples isolated from 5 individual *db/db* mice and 5 age- and gender-matched *db/*+ controls for proteomic analysis. In order to obtain objective and reliable results, we optimized and employed a reproducible, extensive and well-controlled strategy for profiling of the MAM proteome as shown in Fig. 2. All samples were prepared and analyzed in a random order to obviate analytical bias.

**Figure 3:**
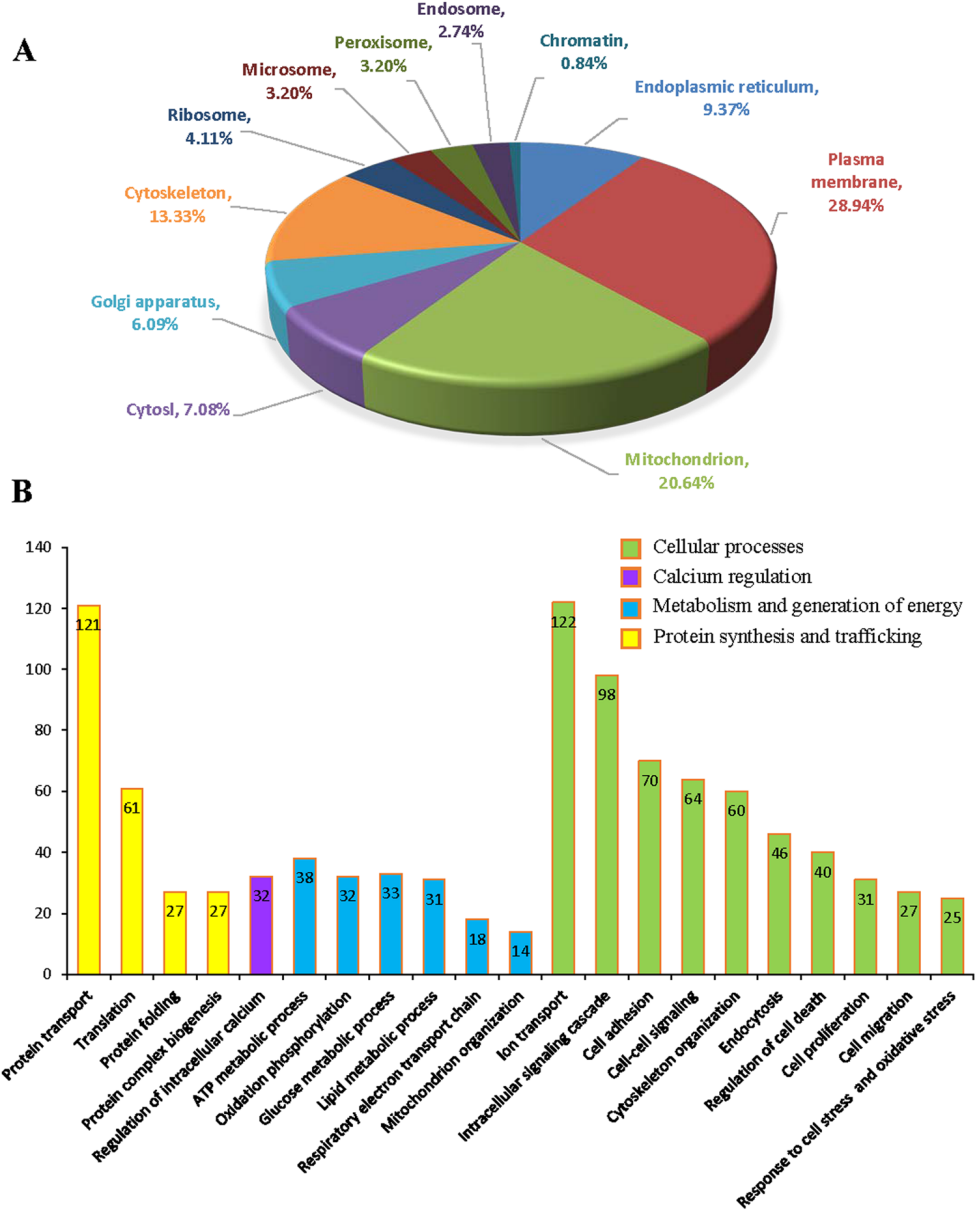
Localization and biological relevance of the mouse brain MAM proteins identified by proteomics. (**A**) Organelle association for MAM proteins determined by annotation. (**B**) According to GO biological processes analysis, 4 major clusters cellular activities including 21 catalogues of biological processes are shown. Processes with a *p* value less than 0.01 were considered significant.

To achieve reproducible, accurate and sensitive protein quantification from the isolated membrane fraction, we employed a newly developed straight forward surfactant-aided-precipitation/on -pellet-digestion (SOD) strategy to for the preparation of MAM samples^23^. This strategy uses a lysis buffer high in detergent composition with extra sonication, allowing exhaustive extraction of proteins, especially for those hydrophobic membrane proteins localized in detergent-resistant MAM domains^24^. Also, unfavorable sample matrix components such as phospholipids could also be completely removed with detergents during organic solvent precipitation. Using this method, 1313 non-redundant proteins were identified in the MAM. Remarkably, 631 out of 1,313 proteins (48.06%) with Gene Ontology (GO) Cellular Component information available belong to either plasma or organelle membrane fraction (Fig. 3A). Additional analysis to predict the number and topology of integral membrane proteins (IMPs) was also conducted and 423 out of 1,313 proteins (32.22%) were predicted to have at least one transmembrane domain (TMD) (see Supplementary Table S2). These results imply the high efficiency in membrane protein retrieval by the usage of our SOD strategy.

Along with additional fractionation and separation methods such as long gradient nano-LC, strong cation exchange (SCX) chromatography and gel electrophoresis, an ion-current-based MS1 quantitative method developed by our lab was selected in this study mainly because of its outstanding quantitative accuracy and precision, high sensitivity in quantification of low-abundance proteins, avoidance of missing value rate^25, 26^. Among the 1,313 proteins identified with high stringency (0.19% peptide FDR; see Supplementary Table S3), confident quantification of ~95% of total proteins (1,239 out of 1,313) was achieved using the aforementioned method, with excellent run-to-run reproducibility (see Supplementary Fig. 1). No missing values on protein level was observed among these quantified proteins, and the average number of peptide quantified for each protein is 9.2 (11,406 peptides quantified in total), which lay a solid foundation for further bioinformatics analysis and biological validation. Under an optimized cutoff of protein expression ratio >1.33 or <0.75 and p-value <0.05, 144 proteins were determined to be significantly altered between diabetic mice (*db/db)* versus the non-diabetic controls (*db/*+) (see Supplementary Table S4 and Supplementary Fig. 2). Table 1 lists the top 20 MAM proteins with most significant changes in MAM from diabetic mice.

**Table 1:** Top 20 significantly up- or down-regulated MAM proteins in diabetes.

Ranks	Protein accession	Symbol	#peptides	Protein names	Ratio (diab/ctrl)	*p* value
**MAM proteins up-regulated in diabetes**						
1	H2A1F_MOUSE	Hist1h2af	5	Histone H2A type 1-F	5.34	0.0385
2	H4_MOUSE	H4-53	7	Histone H4	4.85	0.0398
3	H2B1B_MOUSE	Hist1h2bb	6	Histone H2B type 1-B	4.10	0.0330
4	H31, H32, H33, H3C _MOUSE	H3.1;H3.2 -615; H3.3	2	Histone H3.1, H3.2, H3.3, H3.3 C	3.84	0.0496
5	CAD13_MOUSE	Cdh13	2	Cadherin-13	3.36	0.0036
6	CNRP1_MOUSE	Cnrip1	4	CB1 cannabinoid receptor-interacting protein 1	2.23	0.0024
7	K1C10_MOUSE	Krt10	4	Keratin, type I cytoskeletal 10	2.19	0.0166
—8	TBC24_MOUSE	Tbc1d24	5	TBC1 domain family member 24	2.10	0.0013
9	H10_MOUSE	H1f0	2	Histone H1.0	2.04	0.0304
10	ACBP_MOUSE	Dbi	3	Acyl-CoA-binding protein	2.04	0.0098
11	MOBP_MOUSE	Mobp	2	Myelin-associated oligodendrocyte basic protein	2.03	0.0331
12	HINT1_MOUSE	Hint1	2	Protein kinase C inhibitor 1	1.91	0.0275
13	LAT1_MOUSE	Slc7a5	3	Large neutral amino acids transporter small subunit1	1.89	0.0002
14	SYNE2_MOUSE	Syne2	1	Synaptic nuclear envelope protein 2, Nesprin-2	1.81	0.0369
15	RAB12_MOUSE	Rab12	2	Ras-related protein Rab-12	1.80	0.0465
16	CLD11_MOUSE	Cldn11	4	Claudin-11	1.78	0.0185
17	NOE1_MOUSE	Noe1	1	Noelin (Neuronal olfactomedin-related ER localized protein)	1.77	0.0001
18	MRP_MOUSE	Marcksl1	2	MARCKS-related protein	1.77	0.0169
19	PCSK1_MOUSE	Pcsk1n	5	ProSAAS (IA-4)	1.72	0.0027
20	ERP29_MOUSE	ERp29	5	Endoplasmic reticulum resident protein 29	1.70	0.0161
**MAM proteins down-regulated in diabetes**						
1	AZI1_MOUSE	Cep131	1	Centrosomal protein of 131 kDa	0.14	0.0128
2	P2Y12_MOUSE	P2ry12	3	P2Y purinoceptor 12	0.34	0.0012
3	SKP1_MOUSE	Skp1	6	S-phase kinase-associated protein 1	0.46	0.0091
4	COPB2_MOUSE	Copb2	1	Coatomer subunit beta’ (Beta’-coat protein)	0.47	0.0000
5	DIRC2_MOUSE	Dirc2	2	Disrupted in renal carcinoma protein 2 homolog	0.52	0.0407
6	PPT1_MOUSE	Ppt1	5	Palmitoyl-protein thioesterase 1	0.54	0.0002
7	CHM4B_MOUSE	Chmp4b	4	Charged multivesicular body protein 4b	0.55	0.0004
8	CRYAB_MOUSE	Cryab	6	Alpha-crystallin B chain	0.58	0.0319
9	COPD_MOUSE	Arcn1	3	Coatomer subunit delta	0.58	0.0001
10	ITB2_MOUSE	Itgb2	4	Integrin beta-2	0.59	0.0013
11	PP2AA_MOUSE	Ppp2ca	5	Serine/threonine-proteinphosphatase2Acatalytic subunit alpha	0.61	0.0076
12	DAB2_MOUSE	Doc2	2	Disabled homolog 2 (DOC-2)	0.63	0.0102
13	SE6L2_MOUSE	Sez6l2	3	Seizure 6-like protein 2	0.63	0.0234
14	ACLY_MOUSE	Acly	11	ATP-citrate synthase	0.63	0.0000
15	CNR1_MOUSE	Cnr1	2	Cannabinoid receptor 1	0.64	0.0054
16	COPA_MOUSE	Copa	6	Coatomer subunit alpha	0.64	0.0001
17	KPCG_MOUSE	Prkcg	20	Protein kinase C gamma type	0.65	0.0075
18	ITAM_MOUSE	Itgam	2	Integrin alpha-M	0.65	0.0062
19	ALBU_MOUSE	Alb	5	Serum albumin	0.66	0.0118
20	DOCK7_MOUSE	Dock7	2	Dedicator of cytokinesis protein 7	0.66	0.0230

### Bioinformatic Analysis and Functional Annotation of MAM Proteins

Using DAVID bioinformatics database we analyzed all MAM proteins identified in our study and manually compared the results with previous publications. First, we performed GO analysis to categorize MAM proteins by cellular localizations and biological processes. As shown in Fig. 3A, 123 out of 1313 (9.37%) MAM proteins exist in ER and 271 (20.64%) proteins are associated with mitochondria, while, surprisingly, 380 (28.94%) proteins are destined to the plasma membrane. The enrichment of plasma membrane proteins in the MAM is probably attributed to the dynamic interactions among the ER, plasma membrane and mitochondria^27^. Likewise, it was recently reported that the plasma membrane and plasma membrane-associated membranes are enriched for the ER marker (SERCA2, calreticulin) and MAM makers (e.g FACL, sigma receptor-1 (Sig1R))^24^. Other prominent subcellular locations for the MAM proteins include the cytoskeleton (13.33%, 175 proteins), Golgi apparatus (6.09%, 80 proteins), ribosome (4.11%, 54 proteins), microsome (3.20%, 42 proteins) and endosome (2.74%, 36 proteins). Interestingly, we found small amounts of proteins associated with peroxisome (3.2%, 42 proteins) and chromatin (0.84%, 11 proteins). We speculate that this could be partially attributed to the dynamic interactions between the ER and adjacent organelles such as nucleus, and, as well, intercompartmental translocation of proteins pertinent to pathophysiological processes such as apoptosis^28^. Alternatively, active translocation and trafficking of novel proteins to and from the MAM could contribute to signaling transduction and biogenesis processes, which is yet to be investigated in the future.

### Characterization of Diabetes-associated Changes in MAM Proteome

**Figure 4:**
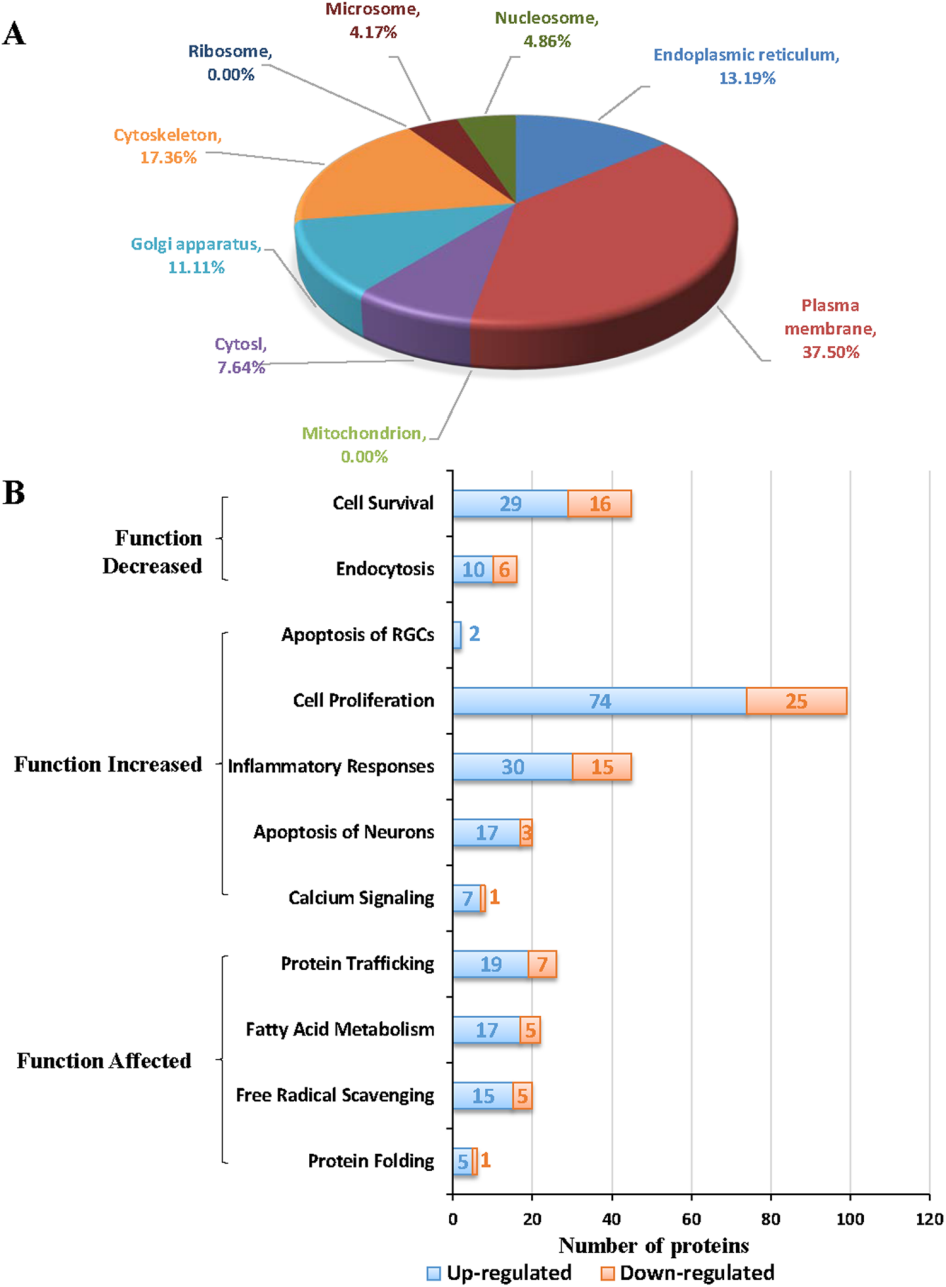
Functional annotation of the MAM proteins that are differentially expressed in *db/db* mice *vs. db/*+ controls. (**A**) The major locations of the MAM proteins that are altered in diabetes. (**B**) Bar graph showing the major categories reflecting the key biological processes pertaining to the pathophysiology of diabetic complications.

To further understand the impact of diabetes on MAM proteome, we performed in-depth analyses to characterize disease-pertinent signaling molecules using both DAVID and Ingenuity Pathway Analysis (IPA) on 144 proteins whose expression levels are significantly altered in diabetic MAM (see Supplementary Table S4). The major cellular localizations of these proteins are plasma membrane (37.50%, 54 proteins), cytoskeleton (17.36%, 25 proteins), ER (13.19%, 19 proteins) and Golgi apparatus (11.11%, 16 proteins) (Fig. 4A). Functionally, these proteins are implicated in biological processes that appear to be closely related to diabetic injury of neurons and blood vessel (Fig. 4B), including cell proliferation (99 proteins), cell survival (45 proteins), inflammation responses (45 proteins)^29^, protein trafficking and folding (32 proteins)^30^, free radical scavenging (20 proteins), neuronal apoptosis (20 proteins), and calcium signaling (8 proteins). These findings suggest that alterations in MAM proteins could contribute to the development of diabetic neurovascular complications.

### Identification and Validation of Key Signaling Pathways and MAM Proteins Altered in Diabetes

**Figure 5:**
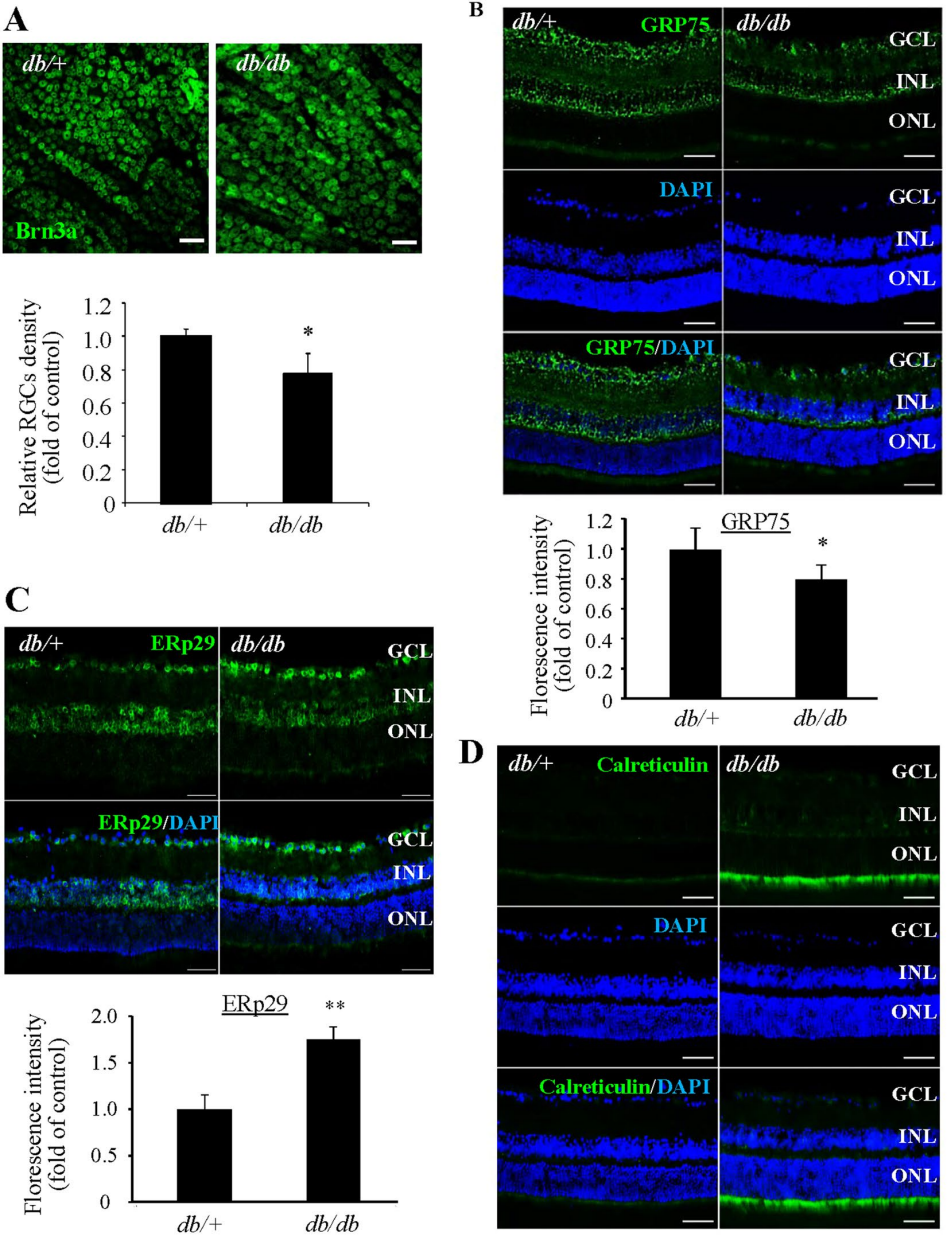
Loss of retinal ganglion cells and decreased retinal GRP75 expression in *db/db* mice. (**A**) Retinal whole mounts were stained for Brn3a to visualize the retinal ganglion cells (RGCs) and examined by confocal microscope. The density of RGCs was decreased in *db/db* mice. Scale bar = 50 μm. Data were shown as mean ± SD, n = 3. *P < 0.05. Student’s *t* test. (**B–D**) Immunostaining showing decreased GRP75 (**B**), ERp29 (**C**), and calreticulin (**D**) in *db/db* mouse retinas and controls. Scale bar = 50 μm for (**B–D**). Images represent results from 3 individual mice in each group. GCL: ganglion cell layer, INL: inner nuclear layer, ONL: outer nuclear layer. Fluorescence intensity was quantified by Image J software and expressed as fold of change relative to control (mean ± SD, n = 3). *P < 0.05. **P < 0.01, Student’s *t* test.

Using immunofluoresence method, we validated the alterations of these pathways in the retina of 15-month-old db/db mice, which present significant degenerative changes in the microvascular system^31^. Further, we found that the number of retinal ganglion cells (RGCs) was significantly decreased compared to db/+ controls (Fig. 5A), indicating a loss of retinal neurons. These results, together with the decreases in absolute brain weight and in brain to body weight ratio in db/db mice, provide further evidence of neuronal degeneration in the central nervous system of diabetic animals^17, 18^. To evaluate the changes of MAM, we performed immunofluorescence labeling to examine the level of MAM marker protein GRP75 in the retina. GRP75 is a major tethering protein that forms a bridge connecting IP3R on the ER and VDAC on mitochondria^8^. We found that the level of GRP75 was drastically reduced in db/db retinas (Fig. 5B), which indicates that the formation of MAM may be altered during long-term diabetes. Next, we examined the cellular expression of ERp29 and calreticulin, two major MAM-located chaperone proteins that are altered in diabetes (see Supplementary Table S4). Consistent with the proteomic results, immunofluorescence study confirmed an increase in ERp29 and calreticulin in db/db retinas compared to controls (Fig. 5C and D).

**Figure 6:**
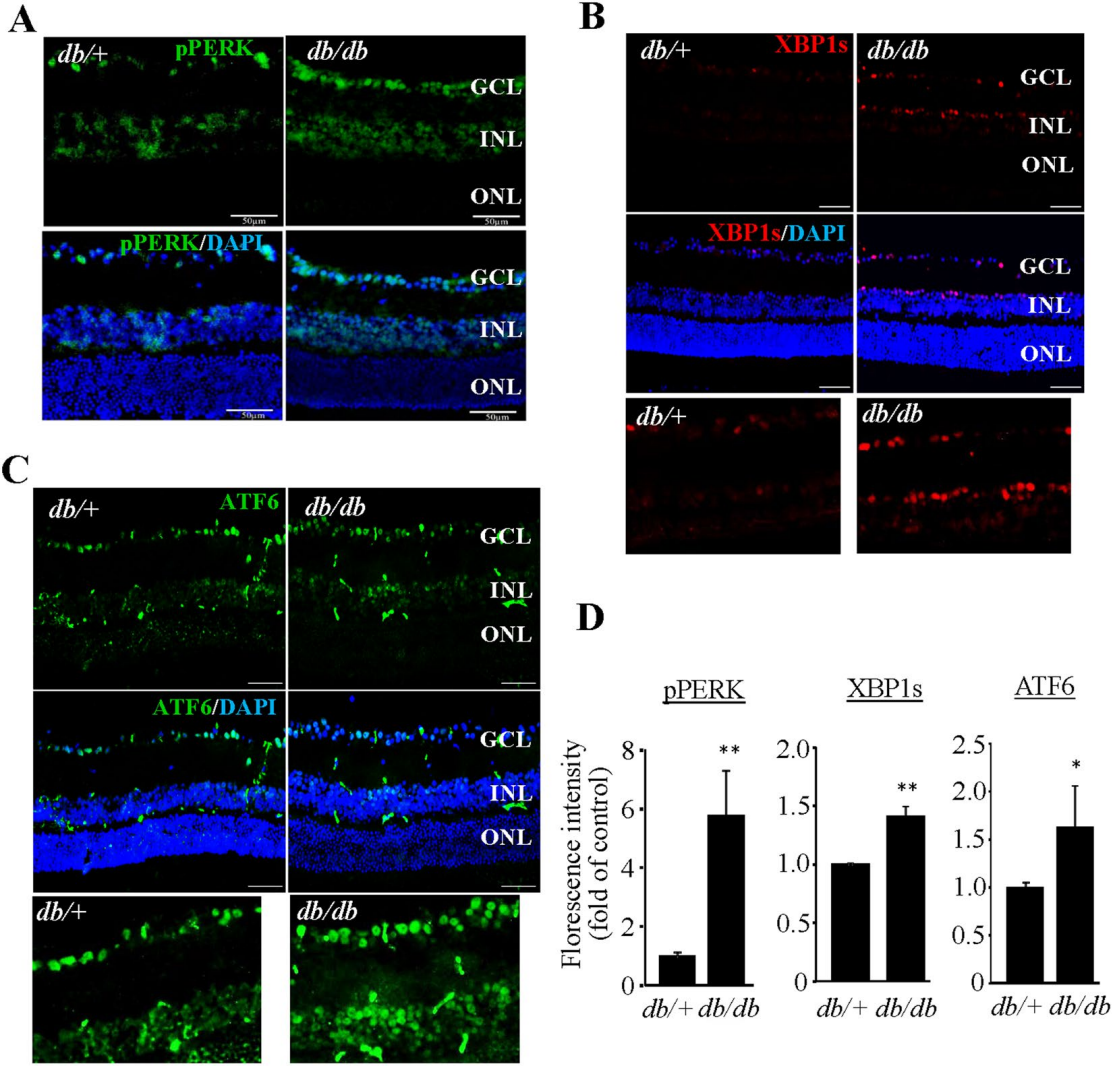
Activation of the UPR and increased level of ER chaperones in the retina of *db/db* mice. (**A–C**) Immunostaining of retinal sections from *db/db* mice and *db/*+ controls of for p-PERK (**A**), XBP1s (**B**), and ATF6 (**C**). Scale bar = 50 μm for (**A**–**C**). Images represent results from 3 individual mice in each group. GCL: ganglion cell layer, INL: inner nuclear layer, ONL: outer nuclear layer. (**D**) Fluorescence intensity was quantified by Image J software and expressed as fold of change relative to control (mean ± SD, n = 3). *P < 0.05. **P < 0.01, Student’s *t* test.

An important finding, though not surprising, is the identification of the UPR as a key pathway in correlation with diabetic changes in MAM proteome. Activation of the UPR has been observed in the brain and retinal tissues from type 2 diabetic animals^32, 33^. Among the three UPR branches, the XBP1 and protein kinase RNA-like endoplasmic reticulum kinase (PERK)/activating transcription factor 4 (ATF4) pathways were more likely to be affected by diabetes^7^. IPA analysis of MAM proteome predicts that XBP1 activation is suppressed, although the level of some individual target proteins is increased (Table 2); in contrast, the function of PERK/ATF4 pathway is significantly enhanced (p = 6.33E-03) (Table 3) in diabetes. Using immunofluorescence labeling of UPR molecules, we confirmed increased expression of pPERK in db/db retinas (Fig. 6A). Notably, recent studies have found that upon activation by reactive oxygen species (ROS)-induced ER stress PERK translocates to the MAM resulting in apoptosis^34, 35^. In addition, we observed strong immunoreactivity against two major UPR transcription factors XBP1s and Activating transcription factor 6 (ATF6) in the nuclei of inner retinal neurons (Fig. 6B and C). These results suggest that enhanced ER stress is perhaps a cause of the changes in MAM proteome in diabetes and is associated with diabetic pathologies. The exact role of the UPR pathways in regulation of the MAM function in diabetes remains elusive.

**Table 2:** Altered MAM proteins involved in the XBP1 pathway.

	ID	Protein names	Prediction (based on expression direction)	Ratio (diab/ctrl)	*p* value
1	ERP29_MOUSE	ERP29	Inhibited	1.70	0.0161
2	CALR_MOUSE	Calreticulin	Activated	1.47	0.0183
3	PRIO_MOUSE	Prion protein	Activated	1.46	0.0078
4	VAMP4_MOUSE	Vesicle-associated membrane protein 4	Inhibited	−1.41	0.0041
5	COPD_MOUSE	Archain Vesicle Transport Protein 1	Inhibited	−1.71	0.0001
6	COPB2_MOUSE	Coatomer Protein Complex, Subunit Beta 2	Inhibited	−2.11	0.0000

**Table 3:** Altered MAM proteins involved in the PERK/eIF2/ATF4 pathway.

	ID	Protein names	Prediction (based on expression direction)	Ratio (diab/ctrl)	*p* value
1	MOG_MOUSE	Myelin Oligodendrocyte Glycoprotein	Activated	1.68	0.0169
2	SERC_MOUSE	Phosphoserine Aminotransferase 1	Activated	1.48	0.0337
3	MBP_MOUSE	Myelin basic protein	Activated	1.41	0.0151
4	MYPR_MOUSE	Proteolipid protein 1	Activated	1.39	0.0184
5	LAT1_MOUSE	L-Type Amino Acid Transporter 1	Activated	1.89	0.0002
6	CALR_MOUSE	Calreticulin	Activated	1.47	0.0183
7	NDRG1_MOUSE	N-myc downstream regulated 1	Activated	1.40	0.0103

The p53 tumor suppressor is a nuclear protein that functions as a regulator of transcription and mediates several biological effects, such as growth arrest, senescence, and apoptosis in response to various forms of stress^36^. Among the 144 MAM proteins altered in diabetes, 21 were identified as p53 target proteins (see Supplementary Table S5). The activation z-score of p53 is 1.605 (p = 5.41E-05), strongly suggesting that p53 activation is implicated in MAM dysregulation in diabetes. In support of this hypothesis, recent studies have demonstrated a causal role of p53 in ER stress-induced cell death^37^ and apoptosis^38^. Importantly, p53 was found physically present at the ER/MAM and promotes apoptosis in a Ca^2^+-dependent manner^39^. These findings point out a potential critical role of p53 in diabetic tissue injury through inducing ER stress and MAM dysfunction.

Additionally, IPA analysis reveals a high activation z-score 2.236 for Aryl hydrocarbon receptor nuclear translocator 2 (ARNT2), a hypoxia-activated transcriptional factor (p = 8.45E-03). Five ARNT2 target proteins were found increased in diabetic MAM (see Supplementary Table S6), among which hypoxia-inducible factor 2 β (HIF-2β) increased by 40%. Under hypoxic conditions, both HIF-2β and ARNT2 are able to form heterodimer with hypoxia-inducible factor 1 α (HIF-1α) and bind to the hypoxia-responsive elements in oxygen-responsive genes, such as vascular endothelial growth factor (VEGF). Activation of the HIF-1α/VEGF pathway has been demonstrated in diabetic retinas^31^. However, the cellular signaling that leads to the activation of HIF-1/VEGF is not fully understood. Our study for the first time indicates a potential role of MAM in upstream regulation of this pathway and may therefore identify new molecular targets in mitigating VEGF-mediated pathologies in diabetes.

### Acute High Glucose Treatment Reduces Mitochondria-ER Contact in Endothelial Cells

**Figure 7:**
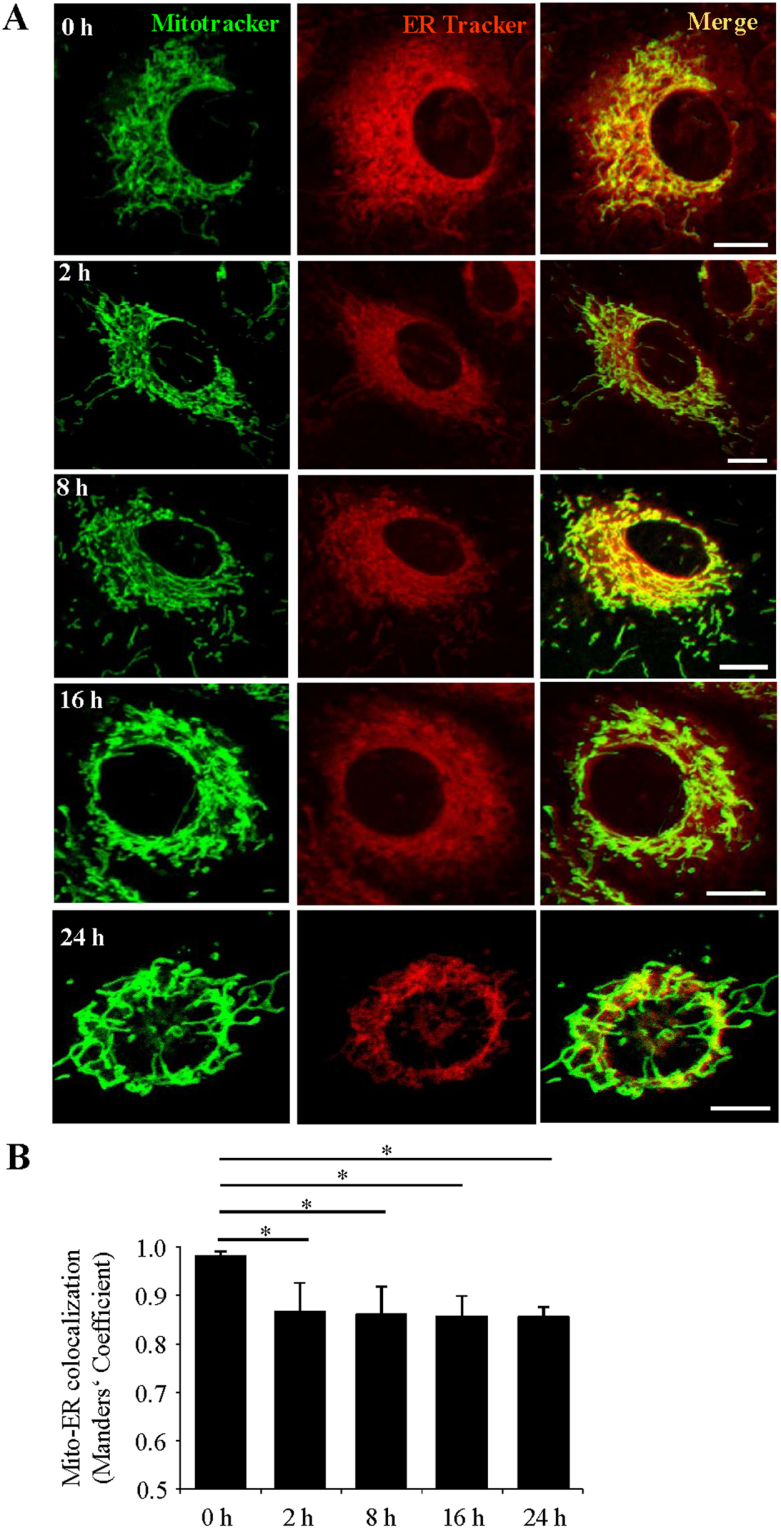
Acute high glucose treatment reduces ER-mitochondrial contact in retinal endothelial cells. (**A**) Representative confocal images of human retinal endothelial cells (HRECs) treated with 25 mmol/ml glucose (HG) for 0 to 24 hours, stained with MitoTracker (green) and ERTracker (red). Scale bars: 10 μm. (**B)** Quantification of the Manders’ coefficient M1 (fraction of mitochondria overlapping with the ER). Mean ± SD; n = 10–15 cells per group). *P < 0.05; one-way ANOVA with Tukey’s post hoc test.

To further evaluate the influence of diabetes on MAM, we conducted an *in vitro* study to examine how mitochondria-ER interaction was altered by acute high glucose treatment. Primary human retinal microvascular endothelial cells (HRECs) were treated with 25 mmol/L glucose for up to 24 h. The time points were selected prior to cells undergoing apoptosis, which was observed at 36 h but not 24 h after high glucose treatment. The mitochondrial and ER contact was visualized using fluorescent probes specifically labeling mitochondria (MitoTracker, green) or ER (ERTracker, red) in live cells by confocal microscopy (Fig. 7A). The colocalization between the ER and mitochondria was quantified using ImageJ (JACoP) software and Manders’ Colocalization Coefficient (MCC)^40–42^ and expressed as the fraction of mitochondria overlapping to the ER (Fig. 7B). Our results show that high glucose rapidly reduces mitochondria-ER contact in endothelial cells.

## Discussion

In the present study, we have identified 1,313 non-redundant proteins in mouse brain MAM with 1,239 quantifiable proteins in both diabetic and non-diabetic MAMs, among which 144 proteins significantly altered in diabetes with ultra-high quantitative confidence. Bioinformatics interrogation of these proteins revealed a diversity of biological processes of MAM such as cell migration, adhesion, proliferation, protein synthesis and trafficking, as well as metabolism and energy generation. Using IPA database, we assigned the significantly altered proteins to 10 major categories reflecting key biological processes pertaining to the diabetes-related vascular and neuronal damages including cell survival and proliferation, apoptosis of neuron, inflammation responses, protein trafficking and folding, calcium signaling, free radical scavenging and fatty acid metabolism. Further, our results showed decreased expression of GRP75, one of the bridging molecules of MAM, in *db/db* retinas. *In vitro*, acute high glucose treatment reduces mitochondria and ER interaction in HRECs. These results suggest that the composition of MAM, as the physiological and biological link between ER and mitochondria, was significantly modified upon diabetic conditions, and this alteration contributes to the pathogenesis of diabetic complications.

Percoll gradient fractionation is the most widely accepted and well-established method for isolation of MAM from crude mitochondria^19, 20^. Using this method, we successfully obtained MAM fractions from both mouse brain and retinal tissues (Fig. 1A). A strong detergent-containing buffer supplied with protease/phosphatase inhibitor was used to ensure that all the MAM proteins were extracted efficiently. MAM, isolated from individual mouse brain tissue, was enriched for GRP78 and GRP94 and was devoid of gross contamination from cytosol (tubulin) and mitochondria (cytochrome-C) (Fig. 1B), indicating high purity of MAM was obtained. The purity of MAM was further endorsed by the identification of the MAM-associated proteins such as GRP78, GRP75, ERP29, ERP44, calnexin, calreticulin, IP3R and VDAC1 with proteomic profiling. The cellular component analysis shows that 30% of the identified proteins are ER and mitochondrial proteins, whilst the proteins found to be located in Golgi apparatus, plasma membrane and cytoskeleton shall not be considered as contamination due to the highly dynamic interaction between the ER and these organelles^27^, which is facilitated by the rearrangement of cytoskeleton^43, 44^.

The first report of proteomic analysis of MAM from mouse brain was published in 2011 and 919 unique proteins were identified using stable isotope labeling with amino acids in cell culture (SILAC) quantification^45^. Later, Poston and colleagues reported 1,212 proteins in the MAM using the LC-MS/MS method^20^. Comparing with these studies, only 37.6% of the MAM proteins identified in our present study were reported previously. This discrepancy may be caused by the difference in sample processing strategy, method and program setting of LC-MS/MS as well as the age (20 days *vs*. 15 months) and genetic background of the mouse strains^20, 45^. Interestingly, like Poston and colleagues^20^, we were unable to locate two previously recognized MAM-enriched proteins Sigma1R and Ero-1 to the MAM by either proteomic or Western blot analysis. Failure in detecting these proteins could be related to certain conditions that are unfavorable for transporting the proteins to the MAM. For example, the enrichment of Ero1α in the MAM depends on the redox state of the ER and is related to oxygen supply^46^. In addition, possible tissue/cell specificity for protein localization and variability resulting from analyses using different analytic tools/antibodies should be taken into consideration. Nevertheless, by employing the state-of-the-art profiling strategies, we have identified a significantly higher number of proteins in the MAM with majority of them being novel proteins. These results have expanded our current knowledge of MAM proteome, and should be conducive for a better understanding of the structural and functional roles of MAM.

Functional annotation of the MAM proteins suggest that MAM is heavily involved in protein transport and translation, protein folding, intracellular signaling cascade, cellular energetics, and calcium homeostasis (Fig. 3B). Among the MAM proteins identified are calcium channel proteins including sarcoplasmic/endoplasmic reticulum calcium ATPase (SERCA), ryanodine receptor (RYR), VDCA, IP3R, and calcium uniporter protein, and calcium-dependent chaperones including GRP75, calnexin, calreticulin, calmodulin (CaM) and CaM kinase II subunits). Our recent study demonstrates that disturbed ER calcium homeostasis causes disorganization of cytoskeleton and disruption of tight junctions in retinal pigment epithelium^47^. In pancreatic β cells, mutation of the ER calcium channel type 2 ryandine receptor (RyR2) led to intracellular calcium leak resulting in activation of ER stress response, mitochondrial dysfunction, and reduced insulin secretion^48^. Disturbance in the ER calcium homeostasis has also been implicated in a variety of human diseases including heart failure, vascular disease, and age-related neurodegeneration^49^. While diabetes significantly increases the risk of cardiovascular and neurodengerative diseases, the mechanisms behind the link of these diseases remains elusive and may involve calcium dysregulation and MAM abnormality. Thus, the potential role of the MAM in regulation of the calcium signaling pertinent to various disease conditions is of great interest and warrants future investigation.

Based on the quantitative changes in MAM proteome, we conducted an in-depth IPA analysis to identify key signaling pathways that may lead to MAM anomalies in diabetes. Among the top activated pathways predicted by the changes in diabetic MAM proteome are the UPR^32, 50^, P53 signaling^51^, and activation of hypoxia-induced genes^52, 53^. These results reveal for the first time a link between diabetes-associated MAM abnormalities and key signaling pathways controlling cellular stress response, apoptosis and hypoxia-related factors.

Among these proteins, one of particular interest is α-basic crystalline (CRYAB), an ER stress-inducible chaperone, which shows over 40% of decrease in diabetes (see Supplementary Table S4). Deficiency of CRYAB has been shown to augment ER stress-induced mitochondrial dysfunction and exacerbates apoptosis of retinal cells^54^. Our results for the first time illustrate the specific location of CRYAB to the MAM. This finding is in agreement with the protective role of CRYAB against ER stress-induced mitochondrial injury, though the exact function of CRYAB in the MAM warrants further investigation.

Interestingly, we found that ERp29 is expressed predominantly in the inner retinal neurons while the increase in calreticulin mainly occurs in the inner segments of photoreceptors. Given the critical function of calreticulin as a Ca^2^+-buffering chaperone in maintaining the ER and intracellular Ca^2+^ homeostasis^55^, the increase of calreticulin may be important for photoreceptor survival and function in diabetic condition. Likewise, ERp29 has been shown to protect against oxidative damage of retinal cells^56^, whether it also plays a protective role in inner retinal neurons in diabetes is yet to be investigated.

One limitation of the present study is not being able to obtain sufficient retinal tissue for MAM isolation or alternatively using brain tissue to validate the proteomic results due to the limitation of sample availability from long-term diabetic mice. Although the brain and retina are interconnected anatomically and display many similarities in characteristics such as the presence of blood-brain barrier and blood-retinal barrier, their differential responses to diabetes and distinct manifestations of diabetic complications should not be ignored^57^. As a proof-of-concept study, we successfully isolated MAM from pooled 20 mouse retinas and confirmed the high purity of the MAM. We are currently optimizing the isolation technique to obtain sufficient retinal MAM for proteomic study. In the meantime, we are working to elucidate the functions of the MAM proteins signaling pathways that are altered in diabetes. For example, we observed that 5 out of 10 MAM proteins that show highest increase in diabetes belong to the histone family (Table 1). In a recent study, histone proteins were also found in the MAM proteome, though the finding was largely ignored in the discussion^20^. We suspect that the presence of histone proteins in the MAM proteome may be related to the post-translational regulation of histone proteins, e.g. degradation through the ubiquitin-proteasome system, which could be upregulated by DNA damage in diabetic conditions^58^. Coincidently, the functional annotation of diabetic MAM proteome suggests the activation of methyl CpG binding protein 2 (MECP2), a transcriptional factor that regulates DNA methylation and transcriptional repression (see Supplementary Table S7). Recent studies have linked increased global DNA methylation with retinopathy in T2DM patients, which could possibly involve mitochondria-derived fatty acids and oxidative stress^59^. These data suggest a possibility of MAM being implicated in regulation of histones and epigenetic modification in normal and disease conditions such as diabetes.

**Figure 8:**
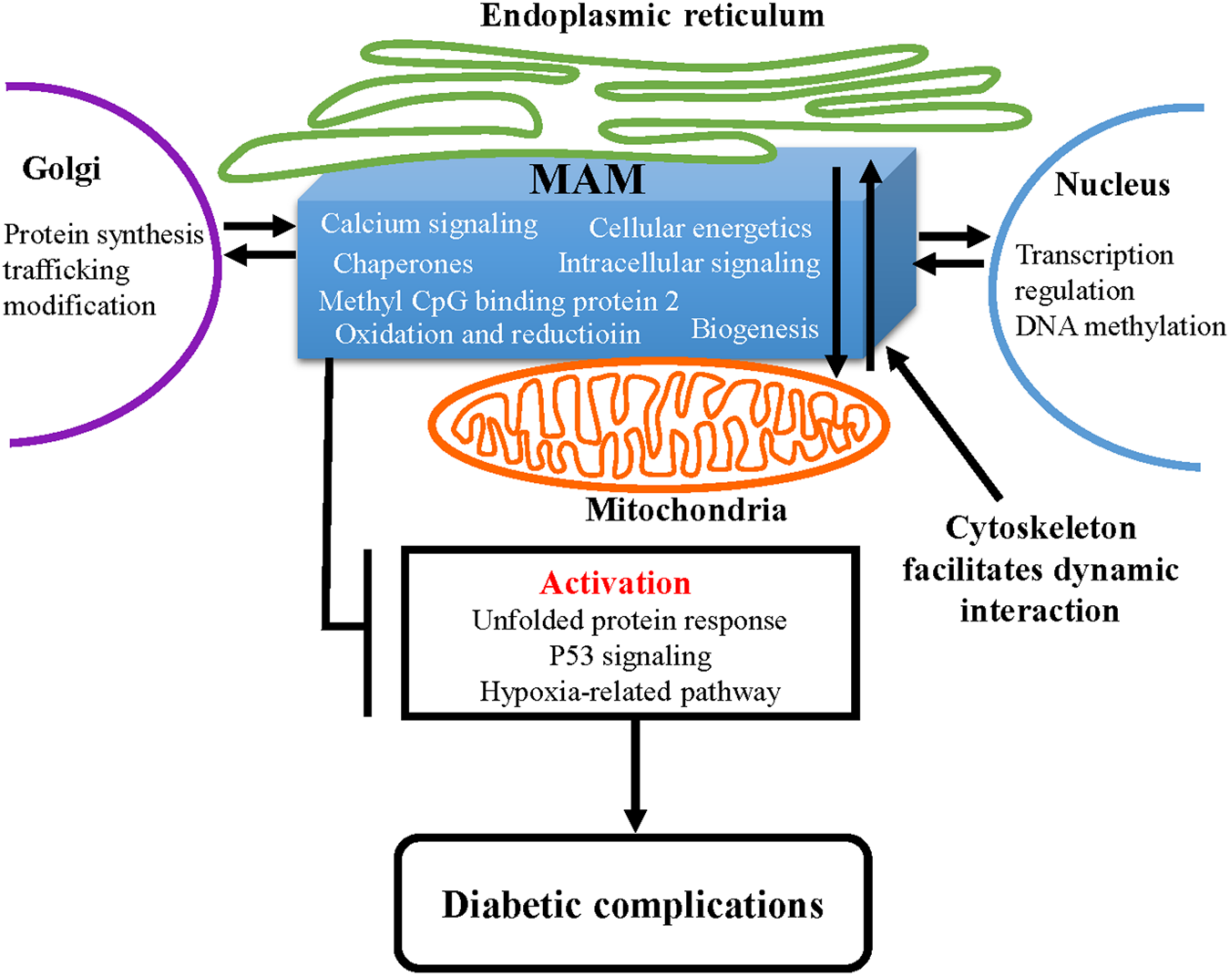
Schematic summary of signaling pathways associated with MAM dysfunction in the pathogenesis of diabetic complications.

In summary, our study presents the first successful and comprehensive proteomic analysis of MAM in a mouse model of long-term experimental type 2 diabetes. Our findings demonstrate significant changes in MAM proteome in diabetic condition and our in-depth bioinformatics analyses have linked the quantitative changes in MAM proteins with key signaling pathways pertinent to the pathogenesis of diabetic complications (Fig. 8). Future studies toward the goal of fully understanding the role of the MAM in regulating the ER and mitochondria associated physiological and pathophysiological cellular events in diabetes will likely uncover new mechanisms for neurovascular injury in the pathogenesis of diabetic complications.

## Materials and Methods

### Animals

*Lepr* ^*db*^ (*db/db*) mice and age- and gender-matched controls (*db/*+) (Jackson Laboratories, Bar Harbor, ME) were used for the study. All animal procedures were approved by the Institutional Animal Care and Use Committees at the University at Buffalo, State University of New York, and in accordance with the guidelines of the Association for Research in Vision and Ophthalmology statements for the “Use of Animals in Ophthalmic and Vision Research”.

### Isolation of MAM, Mitochondria and ER Fractionations from Mouse Brain and Retina

The MAM was isolated following an established protocol (Fig. 1A)^19^. Briefly, tissues were manually homogenized on ice. Nuclei and unbroken cells were pelleted by centrifugation. The supernatant was collected and centrifuged to separate crude mitochondria from microsome and ER fractions. After two washes, the crude mitochondrial fraction was suspended in 2 ml of mitochondrial re-suspension buffer (MRB, 250-mM mannitol, 5-mM HEPES, pH 7.4, and 0.5-mM EGTA), layered on top of 30% percoll medium (225-mM mannitol, 25-mM HEPES, pH 7.4, 1-mM EGTA), and centrifuged at 95,000 × *g* for 30 min. The MAM fraction was extracted from percoll gradient and further purified by centrifugation to remove contaminated mitochondria. Likewise, the pure mitochondria fraction (Mp) was collected from percoll gradient and centrifuge to obtain pellet. All the fractions were flash frozen by liquid nitrogen and preserved in −80 °C until use.

### MAM Protein Preparation and Digestion

The MAM were dissolved in a detergent-containing buffer (50 mM Tris-formic acid (FA), 150 mM NaCl, 1% sodium deoxycholate, 2% sodium dodecyl sulfate (SDS), 2% IGEPAL® CA-630, pH 8.0) plus the protease and phosphatase inhibitor tablets (Roche Applied Science, Indianapolis, IN), and were sonicated for 30 s (non-continuously, 5 s as a burst) using a high-energy sonicator (Qsonica, Newtown, CT). The solution was then centrifuged at 20,000 × g, 4 °C for 30 min, and the supernatant portion was carefully transferred to Eppendorf tubes. Protein concentration was measured by bicinchoninic acid assay (BCA) kit (Pierce Biotechnology, Inc., Rockford, IL). One hundred μg of extracted proteins from every sample were utilized for LC-MS analysis. Reduction and alkylation of proteins were achieved by 30 min incubation with 3 mM tris (2-caboxyl) phosphine (TCEP) and 30 min incubation with 20 mM iodoacetamide (IAM), respectively. Both steps were conducted under 37 °C in darkness with constant vortexing in an Eppendorf Thermomixer® (Eppendorf, Hauppauge, NY). The proteins were then subjected to a unique surfactant-aided precipitation/on-pellet digestion (SOD) procedure. Precipitation of proteins was performed by stepwise addition of 9 volumes of cold acetone with continuous vortexing and incubation at −20 °C overnight. After centrifugation at 20,000 × g, 4 °C for 30 min, the supernatant (containing undesirable constituents which may severely impair the overall quality of MS analysis in the protein mixture, e.g. detergents, components of cellular matrix) was removed and the pellets were rinsed with 800 μl of cold acetone/water mixture (85/15, v/v %) and air-dried. Two phases of enzyme addition were employed for the on-pellet-digestion. In phase 1 (pellet-dissolving phase), 50 µL of Tris buffer (50 mM, pH 8.5) containing trypsin at an enzyme/substrate ratio of 1:40 (w/w) was added to the pellets and incubated at 37 °C for 6 h in an Eppendorf Thermomixer®; in phase 2 (complete-cleavage phase), another 50 µL of trypsin solution was added at an enzyme/substrate ratio of 1:40 (w/w). Then the mixture was incubated at 37 °C overnight (12–16 h) to achieve complete digestion, and digestion was terminated by addition of 1 μl formic acid. Supernatant for individual samples containing tryptic peptides derived from 6 μg of proteins was used for LC-MS analysis.

### Long Gradient Nano-Reverse-Phase Liquid Chromatography/Mass Spectrometry

The nano-reverse-phase liquid chromatography (RPLC) system comprised a Spark Endurance autosampler (Emmen, Holland) and an ultra-high-pressure Eksigent (Dublin, CA) Nano-2D Ultra capillary/nano-LC system. A nano-LC/nanospray setup featuring a low-void-volume and high chromatographic reproducibility was employed. Mobile phases A and B were 0.1% formic acid in 2% acetonitrile and 0.1% formic acid in 88% acetonitrile respectively. Samples were first loaded onto a large inner-diameter trap (300 μm inner diameter × 1 cm, packed with Zorbax 3-μm C18 material) with 1% mobile phase B at a flow rate of 10 μl/min, and the trap was washed for 3 min. A series of nanoflow gradients (flow rate = 250 nl/min) was used to back-flush the trapped samples onto the nano-LC column (75 μm inner diameter × 75 cm, packed with Pepmap® 3-μm C18 material) for separation. The nano-LC column was heated to 52 °C so that both chromatographic resolution and reproducibility were significantly improved. An optimized gradient was utilized to resolve the complex peptide mixture, encompassing the following steps: 3 to 8% B over 15 min; 8 to 24% B over 215 min; 24 to 38% B over 115 min; 38 to 63% B over 55 min; 63 to 97% B in 5 min, and finally isocratic at 97% B for 15 min. Under such chromatographic settings, a peptide elution window of >345 min was achieved, with an average peak with of <30 s and a peak capacity of >580.

An LTQ/Orbitrap-ETD hybrid mass spectrometer (Thermo Fisher Scientific, San Jose, CA) was employed to analyze the identity of peptides in the mixture. An “overfilling” approach, which allowed the reinforcement of MS sensitivity while simultaneously guaranteeing the accuracy and resolution, was applied for peptide detection. The spray tip was rinsed by dripping 50% methanol after every three runs to keep ionization efficiency stabilized. The instrument was operated in the data-dependent product ion mode. One scan cycle included one MS1 scan (m/z 310–2000) in the profile mode at a resolution of 60,000 followed by seven MS2 scans in collision-induced dissociation (CID) activation mode to fragment the seven most abundant precursor ions identified in the MS1 spectrum. The target value for MS1 by Orbitrap was 8 × 10^6^, under which the Orbitrap was meticulously calibrated for mass accuracy and Fourier transform (FT) transmission. The use of a high target valve on the Orbitrap enabled ultra-sensitive detection with no compromise to the mass accuracy and resolution. The dynamic exclusion was enabled with the following settings: repeat count = 1; repeat duration = 30 s; exclusion list size = 500; and exclusion duration = 40 s. The activation time was 30 ms, the isolation width was 3 Da for the linear ion trap (LTQ), the normalized activation energy was 35%, and the activation q was 0.25. Five biological replicates from each biological group (*db/db* versus *db/*+) were analyzed in a random manner.

### Protein Identification and Ion-Current-Based Quantification

Individual raw files generated from MS analysis were searched against the reviewed Mus Musculus UniProt-Swissprot protein database (released on June 2013) with a total of 16616 protein entries using SEQUEST-embedded Proteome Discoverer (PD Version 1.2.0.208, Themo-Scientific). Raw files were imported into PD and DTA files were generated from MS^2^ spectra. The search parameters used were as follows: 25-ppm tolerance for precursor ion mass and 1.0 Da for fragment ion mass. Two missed cleavages were permitted for tryptic peptides. Carbamidomethylation of cysteines and oxidation of methionine were set as fixed and variable modification, respectively. The false discovery rate was detected by the usage of a target-decoy search strategy, in which the sequence database contains each sequence in both forward and reversed orientations and enables the estimation of false discovery rate (FDR). Scaffold software (v4.3.2, Proteome Software, Portland, OR) was used to validate MS/MS based peptide and protein identification based on cross-correlation (Xcorr) and Delta Cn values. The peptide filtering criteria included Delta Cn scores >0.1 and Xcorr scores >1.1, 1.4, 1.7 and 2.5 for singly, doubly, triply, and quadruply charged peptides. Stringent cutoffs for the Delta Cn and Xcorrs scores, plus additional requirement that at least two distinct peptide sequences are needed for identification of a protein, resulted in a considerably low FDR (0.19% at the peptide level). Shared peptides are retained on the identification level, but are further evaluated on quantification level for the congruity of including these peptides for quantification.

SIEVE® software (v2.1.377, Thermo Scientific, San Jose, CA) was used to perform chromatographic alignment and global intensity-based MS1 feature detection/extraction, consisting of: 1) Global chromatographic alignment of LC-MS runs via the application of ChromAlign algorithm. The alignment scores given by SIEVE as well as the intensities of base-peak-ion current were monitored and benchmarked for quality control; 2) Determination of quantitative “frames” based on mass-to-charge (*m/z*) and retention time in the aligned dataset. Only frames with high-quality area under the curve (AUC) with signal-to-noise ratio >10 were picked so that the quantitative reliability was assured; 3) Calculation of ion intensities among all “frames”. The output files were then merged with the spectrum report file exported from Scaffold to link the MS2 fragmentation scans with each “frame” using an in-house developed R package, *IonStarStat* (available at https://github.com/shxm725/IonStarstat). The normalization of ion current intensities, the rejection of outlier peptides with aberrant intensities, and the aggregation of sum ion intensities from “frame” level to protein level were also achieved by *IonStarStat*. The expression ratio for each protein was calculated based on the ion current peak areas of five replicates in both groups.

### Western Blot Analysis

Samples were lysed in radio immune precipitation assay (RIPA) buffer with protease inhibitor cocktail, PMSF and sodium orthovanadate (Santa Cruz Biotechnology, Santa Cruz, CA). Twenty-five μg of proteins were loaded and resolved by SDS-PAGE followed by electro-blotting onto nitrocellulose membrane. The membranes were probed with primary antibodies listed in Supplementary Table S1 and corresponding HRP-conjugated secondary antibodies. The membranes were developed with SuperSignal West Dura Chemiluminescence Substrate (Thermo Fisher Scientific Inc., Rockford, IL) using a Bio Imaging System (Syngene, Frederick, MD).

### Bioinformatics Analysis

Gene Ontology (GO) annotation was performed using the Database for Annotation, Visualization and Integrated Discovery (DAVID) Bioinformatics Resources v6.7 (http://david.abcc.ncifcrf.gov). Biological processes and cellular components assigned by DAVID were manually examined and distributed into corresponding categories^60^. Information on protein networks, disease relevance and signaling pathways was generated through the use of QIAGEN’s Ingenuity® Pathway Analysis (IPA®, QIAGEN Redwood City, http://www.qiagen.com/ingenuity). Prediction of integral membrane protein topology was performed by the TMHMM Server v2.0 (http://www.cbs.dtu.dk/services/TMHMM-2.0/)^61^.

### Immunohistochemistry

Mouse eyeballs were enucleated and fixed in 4% paraformaldehyde. Following sucrose gradient dehydrated process, the eyeballs were embedded in optimal cutting temperature medium (Sakura Finetek Inc, Torrance, CA) at −80 °C. Cryosections (8 μm) were stained with primary antibodies listed in Supplementary Table S1 and corresponding secondary Alexa Flour 488-conjugated goat anti-rabbit or Alexa Flour 488-conjugated goat anti-mouse antibodies. The fluorescence was examined under an Olympus BX53 microscope (Olympus, Tokyo, Japan).

### Retinal Ganglion Cell (RGC) Counting

Mouse eyes were enucleated and fixed with 4% paraformaldehyde. Retinas were carefully dissected, and incubated with anti-Brn3a antibody (1:200, Chemicon international, Inc., Billerica, MA) for 7 days and Alexa Flour 488-conjugated secondary antibody for additional 24 hours at 4 °C. Retinas were flat mounted and examined with a Zeiss LSM confocal microscope. RGC counting was performed using Image J software (National Institute of Health, USA). The density of RGCs in each retina was calculated by averaging the numbers of Brn3a-labeled cells in 8 randomly selected areas (0.45 × 0.45 mm) from four retinal quadrants at 0.5 mm distal to the optic nerve.

### Quantitative Colocalization Analysis of ER and Mitochondria by Fluorescence Confocal Microscopy

Primary human retinal microvascular endothelial cells (HRECs) obtained from Cell Systems, Inc. (Kirkland, WA) were cultured in endothelial growth medium supplemented with EGM-2 SingleQuots (Lonza, Walkersville, MD) in glass bottom culture dishes (MatTek). Confluent monolayer HRECs were quiescent in medium with 2% fetal bovine serum (FBS, Gibco) with half concentration of EGM-2 SingleQuots followed by treatment with 25 mmol/ml glucose for desired time periods. After treatments, HRECs were loaded with 500 nM MitoTracker Green FM (Invitrogen) and 500 nM ER-Tracker Blue-White DPX (Invitrogen) at 37 °C for 30 min. Images of mitochondria and ER were acquired by the Zeiss LSM 510 Meta confocal microscope (Carl Zeiss, Oberkochen, Germany) with a 60x oil immersion objective and processed using NIH’s Image J software. Colocalization of the ER and mitochondria was quantified as Manders’ Colocalization Coefficient (MCC)^41, 42, 62^ using JACoP plugin in 10–15 randomly selected cells per condition in each independent experiment. Auto-thresholds were applied for both channels to select pixels for colocalization analysis. MCCs were then calculated as measure of the fraction of mitochondrial pixels in contact with the ER (with a higher value representing more colocalization).

### Statistical Analysis

Data are expressed as mean ± SD. Statistical analysis was performed using Student’s *t* tests for comparing two groups and one-way ANOVA with Tukey’s post hoc test for comparison of three groups or more. Statistical significance was accepted as *p* < 0.05.

## Electronic supplementary material

Supplementary info

Dataset 1

Dataset 2

Dataset 3

Dataset 4
