# Supplementary material for: Comparative Proteomic Analysis of the Mitochondria-associated ER Membrane (MAM) in a Long-term Type 2 Diabetic Rodent Model
Source: Sci Rep. 2017 May 18;7:2062. doi: 10.1038/s41598-017-02213-1 (PMC5437025; doi:10.1038/s41598-017-02213-1)
Supplement: Supplementary file 1 — Supplementary info [file 41598_2017_2213_MOESM1_ESM.pdf]

Supplementary information

Comparative Proteomic analysis of the Mitochondria-associated ER Membrane (MAM) in a Long-term Type 2 Diabetic Rodent Model

Jacey Hongjie Ma[1, 2, 3 ⊗], Shichen Shen[4, 5, ⊗], Joshua J. Wang [1,2], Zhanwen He[4, 6], Amanda Poon[4], Jun Li[5, 7], Jun Qu[5, 7] and Sarah X. Zhang[*, 1, 2, 4]

1. Department of Ophthalmology and Ross Eye Institute, University at Buffalo, State University of New York, Buffalo, NY, USA

2. SUNY Eye Institute, State University of New York, NY, USA

3. Aier School of Ophthalmology, Central South University, Changsha, China

4. Department of Biochemistry, University at Buffalo, State University of New York, Buffalo, NY, USA

5. New York State Center of Excellence in Bioinformatics and Life Sciences, 701 Ellicott Street, Buffalo, NY, USA

6. Department of Pediatrics, Sun Yat-Sen Memorial Hospital, Sun Yat-sen University, Guangzhou, China

7. Department of Pharmaceutical Sciences, School of Pharmacy and Pharmaceutical Sciences, University at Buffalo, State University of New York, Buffalo, NY, USA

⊗ Jacey Hongjie Ma and Shichen Shen contributed equally to this study.

Corresponding Authors

* Sarah X. Zhang: Departments of Ophthalmology and Biochemistry, University at Buffalo, The State University of New York, Buffalo, NY 14214, Tel: (716) 645-1808

E-mail: xzhang38@buffalo.edu

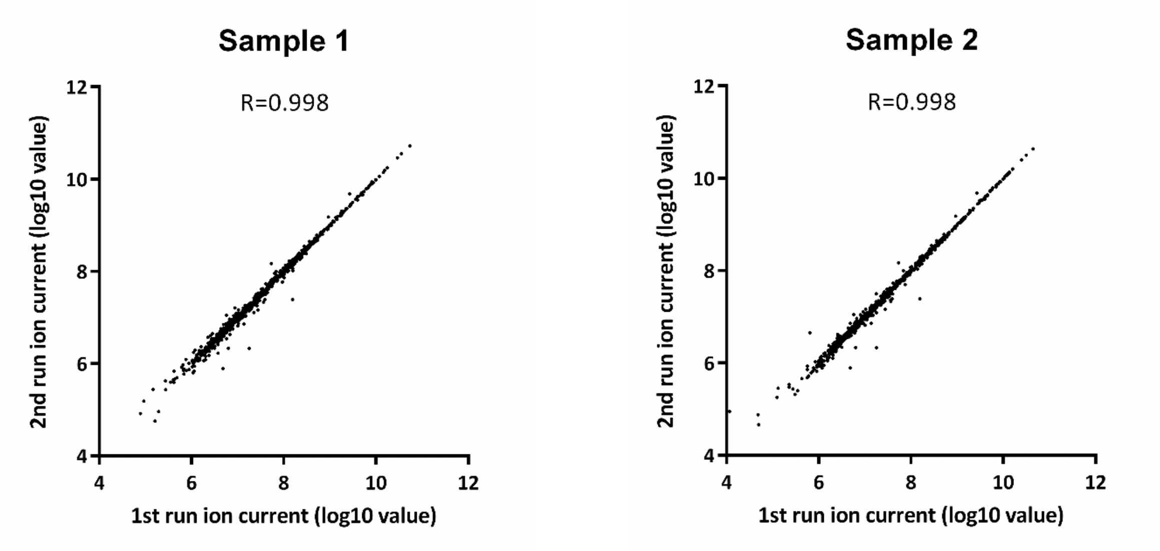

Supplemental Figure 1. Evaluation of the analytical accuracy and precision for quantification of MAM proteome by duplicating each sample. Representative plots of linear correlations between 2 runs.

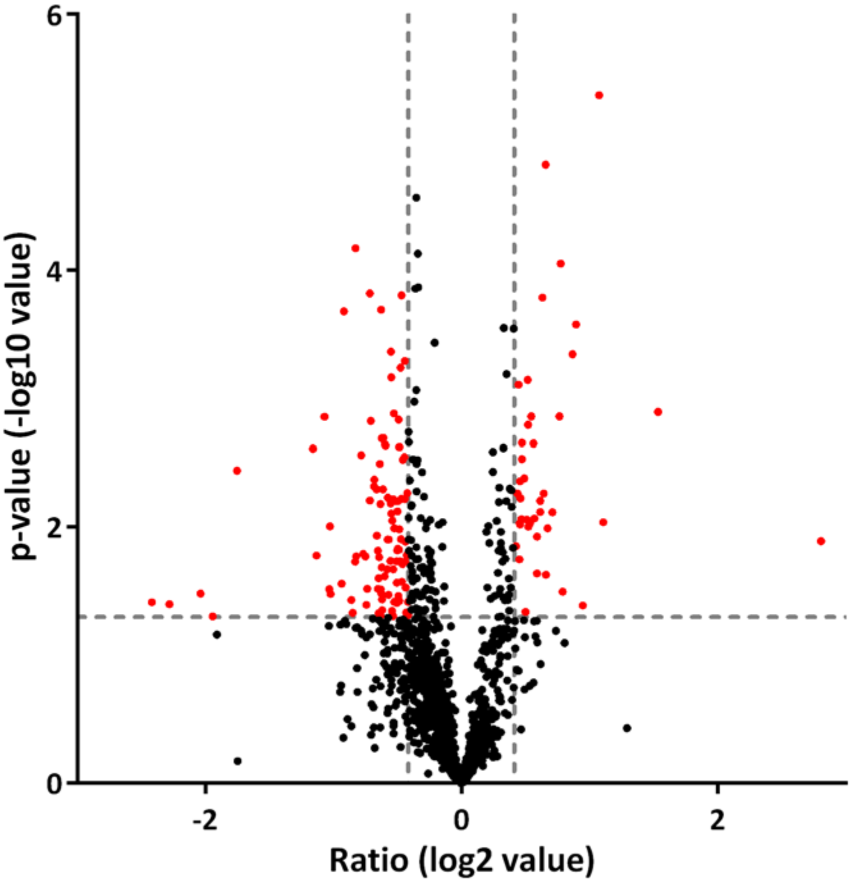

Supplemental Figure 2. The volcano plot for all proteins quantified. Grey dotted lines refer to the cutoff thresholds for significantly changed proteins. 144 out of 1,239 proteins were determined to be significantly changed, as denoted in red.

Table S1. Antibodies applied in immunofluorescence staining (IF) or Western blotting (WB).

Antibody	IF Dilutions	WB Dilutions	Catalog No.	Provider
anti-ATF6	1:50		ab11909	Abcam
anti-Brna3	1:200		ab5945	Chemicon
anti-β-actin		1:10000	ab8226	Abcam
anti-calnexin		1:1000	A01240	GenScript
anti-calreticulin	1:120	1:1000	2891	Cell Signaling Technology
anti-tubulin		1:1000	A01410-40	GenScript
anti-ERP29	1:50	1:1000	ab11420	Abcam
anti-GRP75	1:120	1:1000	3593	Cell Signaling Technology
anti-KDEL		1:1000	ab12223	Abcam
anti-pPERK (Thr 981)	1:50		sc-32577	Santa Cruz Biotechnology
anti-XBP1s (Poly6195)	1:50		619502	BioLegend
anti-Cytochrome C		1:1000		Dr. Luke Szweda (Oklahoma Medical Research Foundation)

Table S5. MAM proteins involved in the P53 pathway.

	ID	Protein names	Prediction (based on expression direction)	Ratio (diab/ctrl)	*p* value
1	K1C10_MOUSE	keratin 10	Affected	2.19	0.0166
2	ACBP_MOUSE	diazepam binding inhibitor (GABA receptor modulator, acyl-CoA binding protein)	Activated	2.04	0.0098
3	PACN1_MOUSE	Protein kinase C and casein kinase substrate in neurons 1	Affected	1.54	0.0328
4	FMO1_MOUSE	Flavin containing monooxygenase 1	Inhibited	1.54	0.0366
5	ASSY_MOUSE	Argininosuccinate synthase 1	Affected	1.54	0.0446
6	PDIA1_MOUSE	Protein Disulfide Isomerase	Activated	1.52	0.0020
7	RAP2A_MOUSE	Ras-Related Protein Rap-2a	Affected	1.47	0.0065
8	PRIO_MOUSE	Prion protein	Activated	1.46	0.0078
9	CLIC4_MOUSE	Chloride intracellular channel 4	Activated	1.44	0.0384
10	UBA1_MOUSE	Ubiquitin-like modifier activating enzyme 1	Inhibited	1.40	0.0150
11	NDRG1_MOUSE	N-myc downstream regulated 1	Activated	1.40	0.0104
12	AP1B1_MOUSE	adaptor-related protein complex 1	Activated	1.39	0.0006
13	TBB3_MOUSE	Tubulin, beta 3 class III	Affected	0.74	0.0054
14	TBB4A_MOUSE	tubulin, beta 4A class IVa	Affected	0.72	0.0022
15	VAMP4_MOUSE	Vesicle-associated membrane protein 4	Affected	0.71	0.0041
16	ALBU_MOUSE	albumin	Activated	0.66	0.0118
17	KPCG_MOUSE	Protein kinase C, gamma	Inhibited	0.65	0.0076
18	ACLY_MOUSE	ATP citrate lyase	Activated	0.63	0.0000
19	PP2AA_MOUSE	Protein phosphatase 2	Activated	0.61	0.0077
20	CRYAB_MOUSE	Crystallin, alpha B	Inhibited	0.58	0.0320
21	PPT1_MOUSE	palmitoyl-protein thioesterase 1	Affected	0.54	0.0003

Table S6. MAM proteins that predict the activation of hypoxia-inducible genes.

	ID	Protein names	Prediction (based on expression direction)	Ratio (diab/ctrl)	*p* value
1	ARNT_MOUSE	Hypoxia-Inducible Factor 1, Beta	Activated	1.40	N/A
2	GTR3_MOUSE	Glucose transporter type 3	Activated	1.34	0.0384
3	MYPR_MOUSE	Myelin proteolipid protein	Activated	1.39	0.0184
4	PGK1_MOUSE	Phosphoglycerate kinase 1	Activated	1.45	0.0493
5	BAG6_MOUSE	Large proline-rich protein BAG6	Activated	1.44	0.0013

Table S7. Proteins that predict the activation of methyl CpG binding protein 2.

	ID	Protein names	Prediction (based on expression direction)	Ratio (diab/ctrl)	*p* value
1	GABR2_MOUSE	Gamma-aminobutyric acid (GABA) B receptor, 2	Activated	1.55	0.0306
2	MBP_MOUSE	Myelin basic protein	Activated	1.41	0.0152
3	DLG4_MOUSE	Synapse-Associated Protein 90	Affected	1.40	0.0024
4	MYPR_MOUSE	proteolipid protein 1	Activated	1.39	0.0184
5	GTR3_MOUSE	Glucose transporter type 3	Activated	1.34	0.0384

Figure 1B

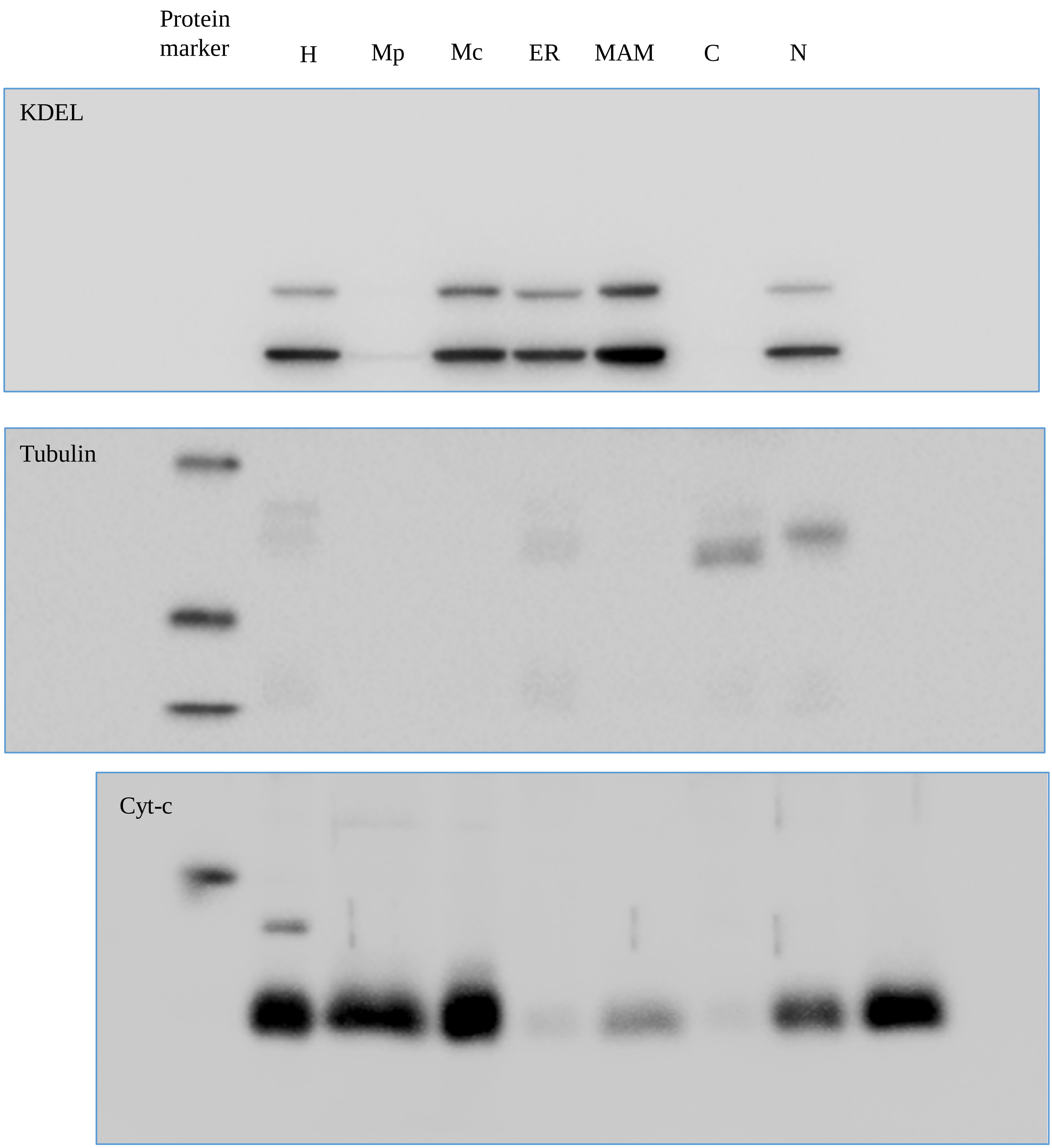

Figure 1C

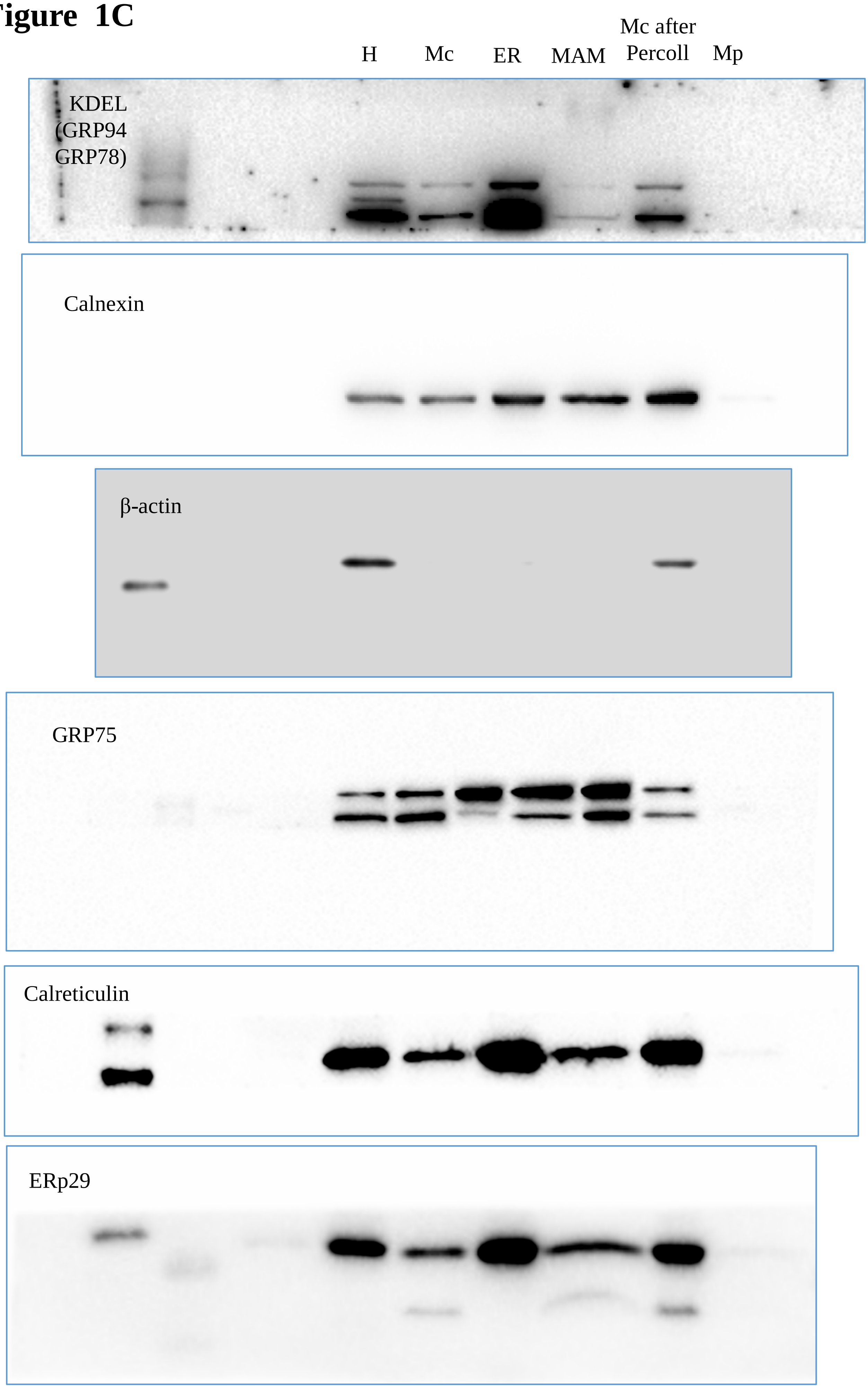
H
Mc
ER
MAM
Mc after Percoll
Mp
KDEL (GRP94 GRP78)
Calnexin
β-actin
GRP75
Calreticulin
ERp29
